# Guidelines and recommendations for radiographer education from the EU-REST project

**DOI:** 10.1186/s13244-025-02104-4

**Published:** 2025-11-03

**Authors:** Jonathan P. McNulty, Francis Zarb, the European Federation of Radiographer Societies, the European Society of Radiology

**Affiliations:** 1 Radiography and Diagnostic Imaging, School of Medicine, University College Dublin, Dublin, Ireland; 2 Department of Radiography, Faculty of Health Sciences, University of Malta, Msida, Malta; 3 EU-REST Consortium, Vienna, Austria; 4 European Federation of Radiographer Societies, Cumiera, Portugal; 5 European Society of Radiology, Vienna, Austria; 6 Department of Radiology, University College Cork, Cork, Ireland; 7 Department of Diagnostic and Interventional Radiology, University Hospital Dubrava, University of Zagreb School of Medicine, Zagreb, Croatia; 8 Division of Cardiovascular and Interventional Radiology, Department of Biomedical Imaging and Image-Guided Therapy, Medical University of Vienna, Vienna, Austria; 9 Discipline of Radiation Therapy, School of Medicine, Trinity Centre for Health Sciences, St. James’ Hospital, Dublin, Ireland; 10 LaTIM, INSERM, UMR1101, University of Brest, Brest, France; 11 Department of Radiation Oncology, Ege University Faculty of Medicine, Izmir, Turkey; 12 Radiation Research Unit, IEO, European Institute of Oncology IRCCS, Milan, Italy; 13 Department of Nuclear Medicine, Cliniques Universitaires St-Luc and Institute for Experimental and Clinical Research (IREC), Université Catholique de Louvain, Brussels, Belgium; 14 Cap Clinic, Servei de Radiofísica i Radioprotecció, Hospital de la Santa Creu i Sant Pau, Barcelona, Spain; 15 Canarian Comprehensive Cancer Center, San Roque University Hospital, Las Palmas, Spain; 16 Fernando Pessoa Canarias University, Las Palmas, Spain; 17 Applied Radiation Therapy Trinity, Discipline of Radiation Therapy, Trinity College Dublin, Dublin, Ireland; 18 Trinity St. James’s Cancer Institute, Dublin, Ireland; 19 Polytechnic University of Coimbra, IPC-ESTESC—Coimbra Health School, Medical Imaging and Radiotherapy, Coimbra, Portugal; 20 H&TRC—Centro de Investigação em Saúde e Tecnologia, Lisbon, Portugal; 21 Budapesti Műszaki és Gazdaságtudományi Egyetem (BME), Budapest, Hungary; 22 National Institute of Oncology, Budapest, Hungary; 23 Department of Health Sciences, European University, Nicosia, Cyprus; 24 Hospital Clínico San Carlos, Universidad Complutense de Madrid, Madrid, Spain

**Keywords:** Curricula, Education and training, Radiation safety, Radiographers, Recommendations

## Abstract

**Abstract:**

Advancements in medical imaging, nuclear medicine, and radiotherapy have significantly improved patient care, while also requiring responsive education and training programmes and continuing professional development (CPD) for radiographers who work across medical imaging, nuclear medicine, and radiotherapy. This article presents findings from the EU-REST (European Union Radiation, Education, Staffing and Training) project with a focus on the evaluation of education and training requirements for radiographers across EU Member States. Evidence-based guidelines to harmonise radiographer education and training and improve safety and quality in medical settings are proposed. The findings highlight the need for standardised, competency-based curricula that align with evolving technologies, safety regulations, and professional responsibilities, together with the importance of integrating radiation safety, quality management, and patient-centred care into curricula. To address accessibility and workforce needs, diverse entry pathways, flexible learning models, and equitable financial support for student radiographers are recommended. Harmonisation of training content, structured clinical placements, and mandatory CPD are also proposed to ensure radiographers remain proficient in emerging technologies such as AI and automation. The findings also underscore the necessity of national accreditation, certification, and licensing systems to maintain high professional standards. Establishing a unified core curriculum at the European level would enhance education quality and ensure compliance with the basic safety standards directive (BSSD). Additionally, postgraduate training opportunities should be expanded to support specialisation and career advancement. By adopting these recommendations, the radiographer profession can cultivate a highly skilled workforce capable of delivering safe, effective, and innovative patient care, ensuring alignment with the future demands of healthcare and technological progress.

**Critical relevance statement:**

The radiographer education and training recommendations developed by the EU-REST project propose a framework to ensure a highly skilled radiographer workforce capable of delivering safe, effective, and innovative patient care, ensuring alignment with the future demands of healthcare and technological progress.

**Key Points:**

- The EU-REST project explored the education and training requirements for radiographers across the EU with a focus on patient safety and quality.
- The development of standardised education and training guidelines for radiographers is essential to ensuring a highly skilled, safe, and effective workforce.
- These recommendations will support the development of competent, adaptable, and research-driven professionals who contribute to the advancement of patient care.

## Introduction

Advancements in medical imaging (MI), nuclear medicine (NM), and radiotherapy (RT) technologies have significantly transformed patient care, expanding the range of procedures available and improving healthcare efficiency through more effective and minimally invasive techniques. Given these rapid technological developments across all three branches of the radiographer profession, MI, NM, and RT, it is essential that radiographers continually update their knowledge and skills to adapt to emerging innovations such as artificial intelligence (AI), demographic shifts (e.g. an ageing population), and evolving disease patterns [1].

The development of education and training guidelines for radiographers must therefore align with an international strategic research agenda that anticipates the impact of technological progress on radiographic practice. Ensuring that radiographers are equipped to provide high-quality, patient-centred care requires integrating research into educational curricula, fostering a culture of continuous learning, and preparing graduates to act as advocates for innovation and professional development [1].

Patient safety, beyond radiation protection, and effective communication are fundamental pillars of professional practice [2]. Education and training programmes should emphasise these aspects, embedding research-driven learning to develop highly qualified radiographers who serve as critical intermediaries between patients and technology [1–3]. Educational institutions must continually adapt their curricula to reflect ongoing technological advancements and evolving professional responsibilities [1, 4].

To ensure radiographers are prepared for lifelong learning, education programmes should incorporate diverse teaching and learning strategies that accommodate different learning styles and practical skill development [5, 6]. Competency assessments should prioritise real-world clinical scenarios while avoiding excessive assessment burdens, a common issue in healthcare education. Additionally, integrating patient and public involvement into radiographer training enhances the relevance and effectiveness of education [1, 4, 7].

Flexibility in training pathways is also essential; consideration should be given to part-time education routes in MI, NM, and RT to support accessibility and career progression. Furthermore, while national policies on trainee remuneration may vary, it is important that student radiographers receive equitable consideration alongside other allied health, nursing, and medical students regarding financial support during clinical training.

By establishing education and training guidelines that reflect these evolving needs, the radiographer profession can ensure its workforce remains prepared to embrace innovation, deliver high-quality care, and contribute to the advancement of healthcare. The European Union Radiation, Education, Staffing and Training (EU-REST) project [8] examined these essential requirements for education and training, with a specific focus on radiation safety across various medical professions, emphasising the need for compliance with the basic safety standards directive (BSSD). According to Article 14 and Article 18 of the BSSD, EU Member States must ensure that practitioners in medical radiological procedures are adequately educated, trained, and continuously informed in radiation protection, with recognition of qualifications and ongoing education [9].

The EU-REST project reviewed the status of education and training for professionals with responsibility for medical ionising radiation. Furthermore, the EU-REST project reviewed the specific education and training needs within each profession, considering new technologies, innovations, and the increasing complexity of medical practices. EU-REST also highlighted the need for harmonised competency-based curricula, funding for training and continuing professional development (CPD), all underpinned by creating a safety culture. It proposed guidelines for education, training, and certification, and stresses the importance of formal assessment/quality assurance of training centres to ensure compliance with legislation and best practices [8, 10].

This article presents the recommendations from the EU-REST project linked to the education and training requirements of radiographers.

## Materials and methods

The education and training requirements for radiographers were explored within the framework of the EU-REST project through a collaborative approach involving a multidisciplinary group. This group, consisting of experts from all relevant disciplines and professions (Radiographers, Radiologists, Nuclear Medicine Physicians, Radiation Oncologists, and Medical Physics Experts [8, 10]), developed guidelines tailored to the unique roles and responsibilities of each professional group.

The foundation of these guidelines was built upon data collected as part of Task 1.2 of the EU-REST project, which included a detailed survey of professional organisations, national societies, government agencies, and regulatory bodies, alongside an extensive literature review of national, EU, and international training standards [8, 10]. The collected data helped to define the key competencies and training requirements necessary for radiographers, considering factors such as workload, technological advancements, and the increasing complexity of medical practices.

To create a systematic and flexible methodology, a baseline framework was established to determine the core education and training needs for radiographers. This baseline serves as a reference point for determining additional requirements based on factors such as evolving job responsibilities, new technologies, and the development of specialised roles. The proposed guidelines of the EU-REST project for radiographers (in MI, NM, and RT) considered current and emerging workforce requirements and were based on current experience and research evidence to ensure both radiation safety and quality of medical radiation applications.

Further details on the full methodological approaches for the EU-REST study have been previously published [8, 10, 11].

## Existing education and training practices

### Entry criteria for radiography education and training programmes

The minimum entry requirement for radiographer education and training is typically set at European Qualifications Framework (EQF) [12] Level 4 or Level 5; however, some higher education institutions (HEIs) also consider alternative qualifications, as well as relevant professional and clinical experience, as meeting entry requirements, while those offering Masters-level programmes for entry to the profession may require a minimum of EQF Level 6. This flexible approach ensures accessibility to individuals with diverse educational and professional backgrounds.

### Structure of radiographer training programmes

In most European countries, radiographer education and training integrates MI, NM, and RT into a single ‘combined’ programme. However, the United Kingdom and Ireland remain exceptions, offering separate educational pathways for MI (inclusive of NM) and RT.

The duration of programmes varies between 2 to 4 years, with total credit allocations ranging from 180 to 240 European Credit Transfer System (ECTS) credits, depending on whether the qualification focuses on a single discipline (MI, NM, or RT) or follows a combined approach. Clinical training constitutes a minimum of 25% of the curriculum, typically equating to 51–60 ECTS; however, there is considerable variation in the structure of clinical education across institutions, with some incorporating simulation-based learning, skills labs, and other innovative training methods [1, 4–6].

The literature reviewed did not provide any specific references to payment during training.

### Curriculum focus: radiation protection, quality management, and safety

While specific hours dedicated to radiation protection and patient safety are not explicitly defined, these core topics are embedded throughout curricula for radiographers [1–5]. Education programmes emphasise competency in radiation safety, quality assurance, and effective patient management to ensure radiographers are well-equipped to practice safely and efficiently.

### Certification and qualification

The majority of radiographer education programmes lead to a bachelor’s degree (EQF Level 6), which serves as the recognised entry qualification for professional practice in MI, NM, and RT across European countries [1, 4, 13]. This level of qualification ensures that graduates meet the necessary academic and clinical competencies to enter the workforce as radiographers, where those qualifying from certain programmes may be eligible to be employed and practice across MI, NM, and RT.

## EU-REST project guidelines and recommendations for radiography education and training in EU Member States

To ensure a high standard of education and training for radiographers across Member States, the following guidelines should be implemented:

### Recommendation 1: entry routes and admission criteria for radiographer education and training programmes

To meet the evolving workforce demands across Member States, it is crucial to prioritise the diversification of entry pathways into radiographer education and training programmes, encompassing MI, NM, and RT. While maintaining traditional admission requirements, alternative entry routes should be developed to accommodate a broader range of candidates. These pathways should include provisions for second-level school leavers who do not meet conventional entry standards, recognition of prior learning for graduates from other higher education disciplines seeking to transition into radiographer education programmes, and individuals with substantial relevant experiential learning, whether healthcare professionals or workers with or without formal third-level qualifications.

Efforts to diversify entry routes should emphasise inclusivity, ensuring equitable access to education and training programmes for individuals from socially disadvantaged backgrounds, those with physical disabilities, and those with specific learning needs. While EQF Level 4 remains the recommended minimum educational standard for entry into programmes, mechanisms should be in place to facilitate access to higher education for candidates who have not successfully completed second-level examinations at EQF Level 4.

Accordingly, the following recommendations are proposed:

- Implementation of diverse entry pathways into radiographer education and training programmes across all EU Member States.
- Development of access programmes to enable all aspiring radiographers to pursue education and training opportunities, regardless of their prior academic achievements or backgrounds.

### Recommendation 2: towards harmonisation of training structure

The European Federation of Radiographer Societies (EFRS) maintains its recommendation that education and training programmes for radiographers be delivered at a minimum of EQF Level 6 (Bachelor’s degree) for entry into all branches of the profession, including MI, NM, and RT [1, 4, 13]. Additionally, the established recommendation for these programmes to comprise a minimum of 180 ECTS credits remains in place [4, 13].

For dedicated programmes focused on a single branch of the profession, it is essential to ensure that the curriculum covers all core theoretical content and clinical competencies relevant to that specialisation. In contrast, combined programmes, integrating MI, NM, and RT, must be designed to encompass the full breadth of core content and clinical experiences across all included disciplines.

Clinical training is a fundamental component of all education and training programmes for radiographers. While variability exists in the proportion of clinical placements within different curricula, it is recommended that clinical placements constitute a minimum of 25% of the total programme ECTS [4]. These placements should be of high quality, providing comprehensive and diverse clinical experiences in recognised and formally approved training centres. Furthermore, these training centres should be subject to regular audits/quality assurance to ensure compliance with educational and professional standards.

Accordingly, the following recommendations are proposed:

- Recognise EQF Level 6 (Bachelor’s) programmes of 180 ECTS as the minimum standard for entry into the radiographer profession across EU Member States.
- Ensure that both dedicated and combined programmes comprehensively cover the core theoretical and clinical components of the relevant branches, with clinical placements comprising at least 25% of total ECTS within approved training centres that are regularly audited.
- Develop diverse, practice-based teaching, learning, and assessment strategies that emphasise real-world clinical scenarios.
- Facilitate part-time programme structures to enhance accessibility and flexibility for students.
- Ensure student radiographers receive equitable treatment in comparison to other healthcare students regarding financial support or payments related to their clinical training.

### Recommendation 3: harmonisation of training content with a focus on radiation protection, quality management, leadership, and safety

Ensuring the effective management of radiation protection and other risks to patients, carers, healthcare professionals, and the public, alongside fostering a strong safety culture, must be a core element of radiographer education and training programmes [1–3, 13]. These principles are essential for the safe and efficient delivery of MI, NM, and RT services, as well as for maintaining high standards of clinical governance.

Research and evidence-based practice must also be central to radiographer education [1, 4, 13]. Programmes should embed research training throughout the curriculum to develop radiographers as critical thinkers and evidence-based practitioners who contribute to advancing the profession and improving patient care [1, 4]. Additionally, fostering a culture of lifelong learning and mandatory CPD is crucial to ensuring that radiographers remain at the forefront of best practices and technological advancements [1, 2, 4, 14].

As the profession continues to evolve, radiographers will increasingly engage with emerging technologies such as automation, AI, augmented intelligence, and robotics [1]. Their ability to critically assess and integrate these technologies into practice will be vital in maintaining patient safety, care quality, and the profession’s role as a human-centred interface between technology and patient care [1, 7].

Furthermore, curricula must emphasise radiation safety and protection, the importance of interprofessional collaboration, and the development of key professional competencies, including effective communication, critical thinking, teamwork, leadership, and ethical practice [1, 2]. Training in quality management and safety, extending beyond radiation protection, should equip radiographers with the skills to actively engage in quality improvement initiatives and enhance service delivery [2, 13].

National regulatory or professional bodies should oversee curriculum development, ensuring alignment with European-level standards and frameworks such as the EFRS EQF Benchmarking Documents for Radiographers [13] and the recommendations of projects such as EURAMED rocc-n-roll [15, 16]. HEIs should be responsible for delivering education and training programmes that are evidence-based, future-focused, and subject to regular review to ensure continued relevance to professional practice [1, 4, 13, 15, 16].

Accordingly, the following recommendations are proposed:

- Establish a standardised minimum core curriculum at the European level, incorporating key topics such as radiation protection, quality management, safety, and related professional competencies across all EU Member States.
- Integrate research training and evidence-based practice as fundamental components throughout radiographer education.
- Emphasise mandatory CPD and lifelong learning, ensuring that all radiographers have opportunities to engage in CPD.
- Develop core curricula that are evidence-based, aligned with recognised frameworks, and regularly reviewed at the national level to reflect the evolving nature of the profession.

### Recommendation 4: towards national certification and registration

Radiographer education and training programmes should lead to an accredited Bachelor’s degree (EQF Level 6), which serves as the recognised entry qualification for professional practice in MI, NM, and RT. Graduates must be registered and licensed to practice independently, with HEIs and training programmes subject to rigorous accreditation processes and professionals required to meet national registration and licensing standards.

Undergraduate/pre-registration programmes should equip radiographers with a high level of professional knowledge, skills, and competencies, ensuring the delivery of safe, high-quality patient care. Additionally, postgraduate education and training opportunities should be available to support radiographers in specialist and expert roles [1, 17]. For example, further qualifications in radiation protection could facilitate radiographers’ progression to roles such as radiation protection officer (RPO) or radiation protection expert (RPE) [18]. Similarly, specialised training in magnetic resonance imaging (MRI) safety could enable radiographers to qualify as Magnetic Resonance Safety Officers (MRSO) [19].

Moreover, it is strongly recommended that radiographer education extends beyond initial certification, with mandatory CPD to ensure that professionals remain up to date with technological advancements, evolving best practices, and emerging research. Such lifelong learning initiatives should align with EFRS recommendations and national regulatory frameworks [1, 4, 14].

Accordingly, the following recommendations are proposed:

- Implement national accreditation systems for radiographer education programmes to ensure consistent quality and compliance with professional standards.
- Establish national certification, registration, and licensing systems for graduates of accredited programmes, with ongoing licensing requirements for practising professionals.
- Recognise the need for postgraduate education and training to support radiographers in specialised and expert roles, such as RPO, RPE, and MRSO.

## Conclusion

The development of standardised education and training guidelines for radiographers across EU Member States is essential for ensuring a highly skilled workforce capable of delivering safe and effective patient care. As advancements in MI, NM, and RT continue to transform clinical practice, radiographers must be equipped with the necessary theoretical knowledge, practical competencies, and professional skills to adapt to emerging technologies and evolving healthcare needs. By implementing the EU-REST project recommendations, the education and training for radiographers can be strengthened to ensure the development of competent, adaptable, and research-driven professionals who contribute to the advancement of patient care and the future of the profession.

## Data Availability

European Commission: European Health and Digital Executive Agency, Analysis on workforce availability, education and training needs for the quality and safety of medical applications involving ionising radiation in the EU—Status and recommendations—Final report, Publications Office of the European Union, 2025, https://data.europa.eu/doi/10.2925/2213975.

## Abbreviations

AI: Artificial intelligence
BSSD: Basic safety standards directive
CPD: Continuing professional development
ECTS: European Credit Transfer System
EFRS: European Federation of Radiographer Societies
EQF: European Qualifications Framework
EU: European Union
EU-REST: European Union Radiation, Education, Staffing and Training
HEI: Higher education institution
MI: Medical imaging
MRSO: Magnetic Resonance Safety Officer
NM: Nuclear medicine
RPE: Radiation protection expert
RPO: Radiation protection officer
RT: Radiotherapy
